# Sexual health and well-being in later life

**DOI:** 10.2471/BLT.24.291576

**Published:** 2024-11-01

**Authors:** Linda McAuliffe, Deirdre Fetherstonhaugh

**Affiliations:** a Australian Centre for Evidence Based Aged Care, La Trobe University Melbourne Campus, Victoria 3086, Australia.

The increase in both the number and proportion of people older than 60 years indicates a global trend of population ageing. The World Health Organization (WHO) estimates that by 2050, the world’s population of those aged 60 years and older will double to 2.1 billion.^1^ Yet although representing a disproportionately large segment of the population worldwide, older people are under-represented in sexual health discourse.

The current WHO definition of sexual health is a state of physical, emotional, mental and social well-being in relation to sexuality, with sexuality defined as a central aspect of being human throughout life.^2^ The definitional emphasis on physical and psychosocial well-being throughout life, including later life, is important.

Despite sustained calls in the literature for greater focus on sexual health and well-being in later life, attention has not substantially increased and the topic remains a persistent policy and practice blind spot.^3^^,^^4^ The sexual health of older adults continues to be notably absent from many government policy documents – both in terms of older people being excluded from sexual and reproductive health and rights agendas, as well as issues of sexual health and well-being not being covered in policies focused on older people.^3^ In the health-care setting, the sexual health and well-being needs of older people are often overlooked due to health workers not having the knowledge or training, or considering the topic to be outside their scope of practice.^4^

## Current evidence

Evidence suggests that many older adults remain sexually interested and capable into later life. A systematic review examining sexual activity in older adults revealed that 30–90% of adults aged 60 years and older reported being sexually active.^5^ This wide range is due to methodological differences including operational definitions of sexual activity. The review included studies conducted in Australia, Belgium, China, Denmark, Islamic Republic of Iran, Malaysia, Kingdom of the Netherlands, Norway, Portugal, Spain, Sweden and the United States of America. Although the younger-old (60–69 years) were found to report more activity than their older counterparts, activity was found for at least some to continue into their 90s.^5^ Types of activity spanned a wide range and included both non-genital and genital activities, with kissing and hugging most frequent, followed by sex with intercourse, and with masturbation one of the least frequently reported activities.^5^

From a health promotion perspective, some literature suggests a relationship between positive sexual activity in later life and well-being, although the evidence is limited. A cross-sectional analysis of data collected as part of the English longitudinal study of ageing found a link between sexual activity and greater enjoyment of life, although the causal direction of this relationship remains unclear. Sexual activity may promote greater enjoyment of life, or experiencing greater enjoyment of life may facilitate being sexually active.^6^ The evidence for potential physical health benefits of sexual activity in older adults also requires further development, with a need to explore underlying mechanisms and causal directions. A study in the United States using national longitudinal data found that partnered sex reduced the risk of hypertension thereby promoting cardiovascular health, but in women only.^7^ The authors noted that sexual activity may not bring uniform health benefits for all.

Evidence also suggests the need for disease prevention in this population. Older adults are more vulnerable to human immunodeficiency virus (HIV) infection and other sexually transmitted infections due to age-related physiological changes (such as thinning of the vaginal wall and weakening immune system) and low condom use.^8^ Results from the Global Burden of Disease (GBD) study 2019 indicate a significant increase in the incidence of HIV among older adults in many GBD regions between 1990 and 2019, including eastern Europe, central Asia and high-income countries in Asia and the Pacific.^8^ Incidence rates of other sexually transmitted infections also increased over this period in North America and Australasia. The highest age-standardized incidence rates of sexually transmitted diseases in 2019 were in eastern, southern and western sub-Saharan Africa.^8^ Increased screening and age-appropriate interventions are clearly needed.

Despite the relevance of sexual health and well-being to older people, the topic remains a neglected area of health care. A rapid review of evidence from 15 systematic reviews from high-income countries regarding later-life sexual health needs indicates that sexual health discussions with older patients is an infrequent practice – although health workers consider such discussions both appropriate and important.^4^ The review found that while health workers believe that older people will raise sexual health concerns, many older people want their primary health worker to raise the topic of sexual health during routine visits.^4^ Other barriers to accessing sexual health care include cultural stigma and/or misperceptions; older people fearing that sexual problems will be dismissed by health workers; and health workers lacking relevant information and knowledge to support later-life sexuality. In contrast, a strong rapport and a welcoming environment were key to open conversations.^4^ The need for strategies to promote open communication on sexual health needs was also highlighted in a review of sexuality studies in African countries. The study found that while sexual activity was viewed as important in old age and common among older people, a new research agenda was required to address knowledge gaps including later life sexual behaviour.^9^

The current body of literature on the topic has many gaps. Queer communities, ethnic minorities and low- and middle-income countries are underrepresented in the research, as are older people living in nursing homes and older people living with disabilities, illnesses or conditions affecting sexual health and well-being.^4^^,^^10^ The literature also lacks specificity in terms of the population included under the term older people, with study samples often spanning a large age range, but with participants most often aged in their 60s, and with more research needed on the needs of those older than 70 years.^10^ More research is needed on the sexual abuse and exploitation of particular populations of older adults, and on the intersectionality of age and gender in relation to sexual health and well-being. Importantly, more evidence is needed on effective interventions to support later-life sexual health,^4^ including interventions to educate older people about sexual health and well-being or help them to access sexual health care, and interventions that provide health workers with the sexual health knowledge they require to deliver best care.

## Future directions

Due to the limited evidence base, much work remains to be done in this space, with high-quality research needed to inform the development of policies that support older adults to experience positive sexual health and well-being.

As a starting point, the global health community needs to acknowledge that sexual activity continues throughout life, and support older adults to experience their sexuality positively and with dignity, with the freedom to engage in safe, consensual and satisfying sexual relationships.^11^ We also need to be aware of the barriers that exist to older people seeking sexual health advice and treatment.

Education can help address some of these barriers. Health workers need – and desire^4^ – education about sexuality and sexual health in later life so that they are equipped with the knowledge required to best treat older people in this area. Such education includes knowledge on the impact of age-related physiological changes such as erectile dysfunction and loss of vaginal lubrication on sexual well-being; sexual dysfunction associated with certain illnesses that are common in older age (such as prostate and breast cancer, cardiovascular disease and diabetes); and the impact on sexual function of the medications and treatments prescribed for these conditions. Education should also cover dementia and sexuality, and later-life sexual orientation, gender identity, gender expression and sex characteristics. As well, education should include developing awareness of the signs of potential sexual abuse and exploitation, as well as of cultural biases, assumptions, ageist stereotypes and traditional gendered norms that may prevent older people, particularly older women, from accessing evidence-based sexual health care.^11^ Education may also lessen any discomfort health workers may feel initiating sexual health conversations with older people, with higher knowledge of health workers found to correlate with higher comfort in discussing sexual matters.^4^

We also need to improve the sexual health literacy of older adults. Doing so is especially important in the context of low condom use and rising rates of sexually transmitted infections including HIV in the older population.^8^ Older adults need information that is accessible and specific, and that refers to further sources of support. A systematic review of psychological interventions for sexual health in older age^12^ suggests that educational and cognitive-behavioural approaches seem to be effective for promoting sexual health in older age; however, further research is needed to confirm these findings. The possibility of novel digital interventions that allow older adults to access support from their own home hold particular promise and warrant exploration. Lastly, older adults may benefit from support to have conversations about sexual health and well-being with their intimate partners.

## Conclusion

The global health community needs to make older adults visible in both sexual health campaigns and government and health-care policies. In addition, we need to include the sexual health of older adults in longitudinal studies of ageing and account for them in population-based studies of sexual health. Crucially, we need to research underrepresented subgroups within the older population and design effective interventions to ensure that every adult realizes full sexual health and well-being, irrespective of their age.
